# Generative AI in Precision Nutrition: A Review of Current Developments and Future Directions

**DOI:** 10.3390/nu18060938

**Published:** 2026-03-17

**Authors:** Lubnaa Abdur Rahman, Vasileios Dedousis, Ioannis Papathanail, Rooholla Poursoleymani, Maria Kafyra, Ioanna Panagiota Kalafati, Stavroula Georgia Mougiakakou

**Affiliations:** 1 Graduate School for Cellular and Biomedical Sciences, University of Bern, 3012 Bern, Switzerland; 2 ARTORG Center for Biomedical Engineering Research, University of Bern, 3008 Bern, Switzerlandstavroula.mougiakakou@unibe.ch (S.G.M.); 3 Department of Nutrition and Dietetics, School of Health Science and Education, Harokopio University of Athens, 17676 Athens, Greece

**Keywords:** precision nutrition, personalized nutrition, generative artificial intelligence

## Abstract

**Background:** Precision nutrition (PN) aims to personalize dietary guidance by accounting for inter-individual variability across biological, metabolic, lifestyle, and environmental factors influencing nutritional needs and health outcomes. While traditional Artificial Intelligence (AI) has advanced nutritional research through systems like automated dietary assessment, these models often operate rigidly. Generative AI (GenAI) introduces the capacity for adaptive interventions for enhanced PN. However, the scope and maturity of its applications remain insufficiently characterized. **Objective:** This review examined original works applying GenAI in PN, focusing on application, methodology, and limitations. **Methods:** A systematic search was conducted in PubMed, ACM Digital Library, and Scopus. Inclusion criteria focused on original works deploying GenAI models in PN contexts. Included works were further formally assessed based on data used, validation, transparency, bias, and security and privacy. **Results:** 21 eligible studies were identified, all published after 2024. The literature indicated a surge in large language model-based systems for personalized dietary recommendations, followed by applications in data foundation building and food effect understanding. A recurrent limitation was questionable evaluation on synthetic data and hallucinations, necessitating a human-expert-in-the-loop, especially in high-stakes clinical settings. Additionally, only 4 of 21 reviewed studies incorporated biological content or biological inputs, and fewer approached biologically grounded PN within implemented personalization workflows using metabolic and/or genomic variables. **Conclusions:** Although GenAI research in PN is expanding rapidly, most applications remain personalized at a user-preference level rather than including biological determinants. The need for standardized reporting, stronger genome-informed modeling, and consistent human-in-the-loop validation protocols is further highlighted to advance towards holistic PN.

## 1. Introduction

Diet is a major determinant of global health: unhealthy dietary patterns are among the leading modifiable drivers of non-communicable diseases [1,2]. Yet most dietary guidance remains grounded in population-level recommendations that insufficiently capture marked inter-individual variability in metabolic responses, nutritional requirements, and real-world feasibility [3]. This gap has accelerated the shift toward precision nutrition (PN), aiming to address a fundamental limitation of conventional nutrition paradigms: the inability to explain why the same dietary exposure can produce markedly different health outcomes across individuals. In this review, to ensure conceptual coherence, we operationally define PN as a comprehensive, multi-dimensional framework integrating both objective, mechanistic biological determinants (e.g., genotype, metabolomic profiling, microbiome features, clinical biomarkers) and subjective, contextual factors (e.g., lifestyle behaviors, user preferences, cultural constraints) to deliver tailored guidance [4]. While personalization often defaults to contextual layers, a complete PN strategy requires the synthesis of both behavioral inputs and biological multi-omics to drive targeted, evidence-based interventions to support more individualized strategies rather than population-level guidance. Specifically, they have been applied both for health optimization and health-related behavior change [5,6]. Additionally, they aid in disease prevention, utilizing stratification, early detection of irregularities, and prognostic monitoring via repeated, high-resolution data, like wearables and digital dietary tools [7,8,9]. Furthermore, they have been applied to disease management, addressing individual variability driven by genetic and microbiome differences to improve outcomes in complex chronic conditions, offering a more effective alternative to one-size-fits-all guidelines [10,11]. Genome-, microbiome-, and/or multi-omics-based PN technologies are expanding rapidly, underscoring both the opportunity and the urgency for frameworks that translate biological heterogeneity into actionable, safe dietary guidance [12].

However, PN’s impact is fundamentally constrained by the quality of dietary exposure measurement [13,14]. Realizing the full potential of PN requires a shift from the traditional, manually curated, and static decision frameworks towards computational systems designed to operate at a bigger scale. PN increasingly relies on heterogeneous, high-dimensional data across many modalities, but conventional statistical models and rule-based pipelines struggle to integrate and reason over this complexity for adaptive, longitudinal, personalized recommendations. This mismatch has positioned Artificial Intelligence (AI) as a central enabler in nutrition research, which refers to computer systems designed to perform tasks that typically require human intelligence, such as learning, pattern recognition, reasoning, and decision-making [15]. Historically, AI in nutrition has focused on automating labor-intensive processes such as dietary assessment, where systems utilize deep learning models to streamline the pipeline into processes of, for example, food segmentation, recognition, volume estimation, and nutrient estimation [16,17,18]. While these advancements have achieved performance parity and, in some cases, superior performance [19,20] compared with traditional manual methods, substantially reducing burdens and improving scalability, they remain largely static and struggle in real-world settings [21,22]. Such traditional models inherently map predefined inputs to fixed outputs, lacking the capacity for complex reasoning, transparent explanation, or adaptive user interaction [18,23,24,25].

Generative AI (GenAI), a subfield of AI, focuses on models that can synthesize new content, including text, images, and tabular data, among others, based on learned representations [26]. The technical landscape of GenAI models includes diverse architectures ranging from variational autoencoders (VAEs) [27] to generative adversarial networks (GANs) [28], diffusion models [29], and large language models (LLMs) [30]. In nutritional sciences, this capability paves the way for context-aware, personalized interventions tailored to specific, ever-evolving user needs. VAEs have been used to predict intake levels and recognize food data [31,32]. GANs and diffusion models have been critical for visual tasks like synthesizing realistic food images, to, in turn, help train more robust systems for estimating portion sizes and tracking dietary intake [33,34]. The most notable advancement is the rapid integration of LLMs for healthcare management driven by their reasoning ability, enabling the provision of expert nutritional guidance, synthesis of diverse health data, while offering adaptive interactions that collectively facilitate long-term management of health problems [35,36,37,38,39,40,41,42].

**Figure 1:**
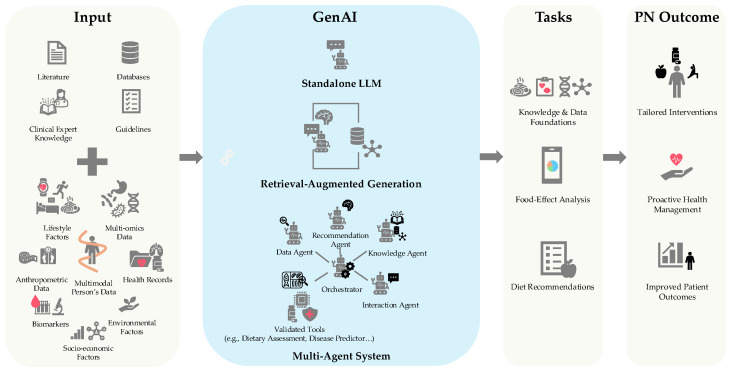
Application of Generative Artificial Intelligence (GenAI), specifically large language models (LLMs), in precision nutrition (PN).

While promising, multiple bodies have underscored a critical limitation: LLMs, off the shelf, are ill-suited for tasks requiring strict arithmetic precision, safety verification, or adherence to rigid clinical protocols and are prone to hallucinations [43,44,45,46]. As such, research is transitioning toward verifiable architectures to constrain LLM output in factual evidence, which is often coined as Retrieval-Augmented Generation (RAG) [47,48]. RAG can be formally defined as a process where the LLM acts like an open-book student, looking up reliable information from a verified external library before it answers a question to ensure its facts are correct. This is being done by leveraging structured external data, such as Knowledge Graphs (KGs), which are digital maps of information that connect different concepts, like recipes, ingredients, and allergens [49,50]. While works have shown efficacy in applying RAG for context-aware nutrition-related recommendations [49], they still struggle at a larger scale and fail to reflect real clinical workflows [51,52]. Recognizing that retrieval alone is insufficient for holistic care, modern frameworks are expanding into multi-agentic systems (MAS) where LLMs function mainly in the tasks of generation and orchestration to delegate specific tasks to validated tools to enhance reliability [53,54,55,56,57,58]. Illustrating this approach, Figure 1 demonstrates an LLM-powered ecosystem for PN, taking as input clinical knowledge, validated databases (DBs), guidelines, and corpora, along with multimodal person’s data to drive tailored health interventions and improved patient outcomes.

Despite the observable surge in GenAI applications in nutrition as laid out previously, a preliminary survey suggests that current literature reviews remain heavily concentrated on predictive and analytical domains, such as dietary assessment, clinical decision support, and disease risk screening [59,60,61,62]. While emerging reviews have begun to outline opportunities for LLMs in clinical settings [63,64], they indicate that only a small proportion of studies utilize generative models for active recommendation and health optimization tasks [65]. However, existing reviews fail to truly capture the specific intersection of GenAI and PN; they tend to focus either broadly on traditional AI in PN or GenAI in general nutrition, leaving the specific intersection of PN and GenAI unmapped. By focusing directly on this intersection, this review clarifies how GenAI is reshaping PN toward more adaptive, explainable, and context-aware forms of guidance. Crucially, by applying this operational definition, we aim to evaluate the extent to which current GenAI systems achieve a holistic PN approach, balancing both user context and biological grounding. It highlights the opportunity for GenAI to advance PN from predominantly predictive work toward truly proactive and personalized intervention and further draws on this to highlight where explorations remain limited and what advances are still needed to shape the path forward in PN meaningfully.

## 2. Materials and Methods

### 2.1. Search Strategy

To identify studies on the application of GenAI in precision and personalized nutrition, a structured literature search was conducted. PubMed, Scopus, and the ACM Digital Library were searched for publications spanning from 1 January 2021 to 31 December 2025. The five-year window was selected because, prior to 2021, applications were predominantly confined to predictive analytics and rule-based systems, with generative architectures not yet established within PN research.

To establish the conceptual boundaries of this review, the search strategy combined keywords using two distinct clusters. The first frames GenAI as a methodological and computational tool. To actively prevent terminology bias and ensure the capture of earlier GenAI architectures that predated the widespread use of the umbrella term “GenAI”, specific models such as VAEs, GANs, and diffusion models were explicitly included in the search. This cluster also incorporated LLM, implementation paradigms (e.g., conversational AI, RAG, multi-agent systems), and prevalent applications (e.g., ChatGPT, chatbots) to capture diverse deployment philosophies and delivery mechanisms. The second cluster targeted the PN domain, combining dominant umbrella terms (e.g., personalized nutrition) with genetic and omics terms (e.g., nutrigenomics, nutrigenetics). Altogether, a comprehensive search string combining the following keywords was formulated:
*(“generative AI” OR “GenAI” OR “large language model” OR “LLM” OR “multi-agent system” OR “conversational AI” OR “variational autoencoder” OR “VAE” OR “generative adversarial network” OR “GAN” OR “diffusion model” OR “RAG” OR “ChatGPT” OR “Chatbot”) AND (“precision nutrition” OR “personalized nutrition” OR “individualized nutrition” OR “nutrigenomic” OR “nutritional genomic” OR “nutrigenetic”)*

These were used to run targeted searches in all three DBs. Searches were restricted to the title and abstract fields to increase precision and reduce irrelevant retrievals. First, duplicates were manually removed based on extracted titles across the DBs. This was followed by a full-text review of studies that satisfied all inclusion criteria and did not meet any exclusion criteria.

### 2.2. Inclusion Criteria

- Original research: experimental, computational, or methodological using GenAI methods.
- Applied GenAI methods directly to precision or personalized nutrition. To map the full landscape of current GenAI applications, we did not differentiate between varying degrees of personalization (e.g., mechanistic and biologically driven versus preference- or contextual-driven) during the inclusion process.
- Published between 2021 and 2025.

### 2.3. Exclusion Criteria

- Used non-GenAI computational methods only.
- Applied solely to nutrition or addressed nutrition, but lacked PN-specific components.
- Lack of proof and explanation demonstrating substantial PN and GenAI contributions.
- Involved non-human subjects.
- Reviews, proceedings, or font matter.
- Not open access, not even with institutional access.
- Had been retracted.
- Were duplicates among PubMed, Scopus, and the ACM Digital Library.

### 2.4. Quality Assessment

Due to substantial heterogeneity in studies, evaluated GenAI works, and reported performance metrics, a meta-analysis and direct comparison were not feasible; therefore, a narrative synthesis approach was used. Although established frameworks [66,67,68] provide guidance for prediction models and clinical AI trials, there was no direct way to apply these standards to works examined in this review. Therefore, based on these, we formalized the quality assessment of each work by reporting on the following five dimensions: Data Type (DT, i.e., real-world or synthetic), Validation Source (VS, i.e., automated, expert reviewers, or user), Lack of Transparency and Reproducibility (LoTR), Risk of Bias (RoB), and Security and Privacy Concern (SPC). To address deployment architecture and provide study-specific evidence, we explicitly distinguish these two concepts within SPC: security refers to the technical safeguards protecting data from unauthorized access, while privacy, in this context, provides governance on how personal data is used, processed, transmitted, and stored within the proposed architecture. Broader compliance and policy governance models were not assessed as they were absent across all the reviewed works. For VS, automated: quantitative evaluation without human judgment, expert reviewers: output assessment by domain experts (e.g., clinicians), human annotator: non-expert or unspecified-credential raters, and user: evaluation with end users (e.g., usability/satisfaction). To ensure objectivity, two authors evaluated each study across the criteria of LoTR, RoB, and SPC, scoring them as defined formally in Table 1, and in case of disagreement, a third author provided a consensus.

## 3. Results

### 3.1. Included Works

**Figure 2:**
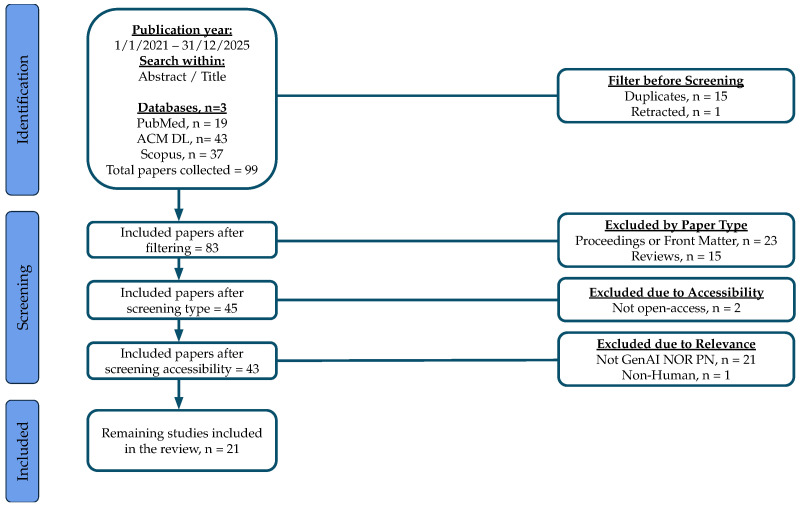
Flowchart of the selection process from PubMed, Scopus, and the ACM Digital Library, resulting in 21 total papers.

Starting with 99 publications identified through the previously mentioned search query, we followed the systematic screening process illustrated in Figure 2. After removing 15 duplicates and 1 retracted work, we excluded 23 proceedings or front matter sections, 15 reviews, 2 non-open-access articles, and 21 papers unrelated to GenAI or PN. Finally, 1 non-human study was removed, resulting in a final selection of 21 English-language works. All exclusions were done manually by going through titles, abstracts, and full text wherever needed.

**Figure 3:**
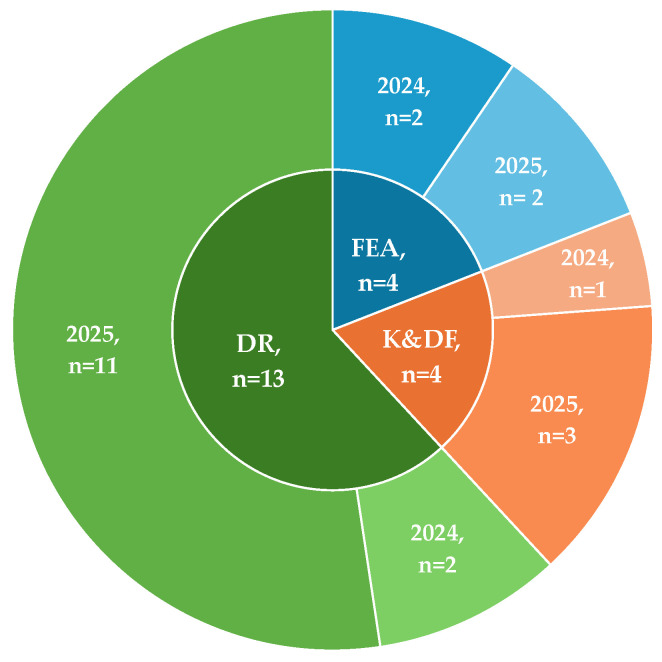
Distribution of papers mapped by precision nutrition application and year. K&DF: Knowledge and Data foundations, FEA: Food-Effect Analysis, DR: Diet Recommendations.

For enhanced organization, we mapped each included study to one of the three main aspects of personalized and PN that their GenAI application primarily represented. The first group consisted of studies categorized under Knowledge and Data Foundations (K&DF), which used GenAI to create knowledge structures or bases that organize nutrition-related information. The second category included Food-Effect Analysis (FEA). Studies in this group leveraged GenAI mainly for evaluating foods’ suitability and their effects with respect to an individual. The final category was Diet Recommendations (DR), which used GenAI to generate or support personalized diet-related recommendations. Even though our search window spanned from 2021 to capture early developments, no eligible papers appeared prior to 2024, indicating that the intersection of GenAI and PN is a genuinely new and rapidly growing area, distinct from earlier traditional AI applications. The absence of older works is not an artifact of terminology bias; our search strategy successfully retrieved publications spanning the entire search window, including those utilizing “older” GenAI methods such as GANs and VAEs. However, during the screening process, these pre-2024 retrievals and papers focusing on VAEs and GANs, with the exception of one, were excluded as they did not meet our predefined inclusion criteria. This is eventually reflected in Figure 3, which demonstrates the distribution of the 21 studies across these three categories and their spread by publication year.

We further summarized the key findings of each included work in Table 2, which lists all 21 studies grouped by K&DF, FEA, and DR alongside their authors and brief statements of their main contributions. To address methodological heterogeneity and support more transparent interpretation of the review’s conclusions, Table 1 was also expanded to include a structured appraisal of each study across the five dimensions and detailed criteria, as defined in Section 2.4.

### 3.2. Reviewed Works

#### 3.2.1. GenAI for Knowledge and Data Foundation

A persistent challenge in PN is the limited size of datasets, especially from human cohorts, required to train AI models effectively. To address this, Xiangyu & Hao [69] developed a generative solution using a Wasserstein GAN with Gradient Penalty (WGAN-GP) to synthesize realistic endurance, in this case rowing performance, and nutritional supplementation response data. By adding high-quality synthetic data to their training set, they significantly improved the predictive accuracy of their recommendation engine for carbohydrate–protein supplementation. The inclusion of this synthetic data significantly reduced the model’s prediction error, confirming that WGAN-GP could replicate and enhance real data patterns in environments with limited information. The application demonstrated how GenAI functioned as a predictive modeling tool in data-scarce settings in elite sports, although the narrow cohort scope (restricted to male endurance athletes) was a possible risk of bias raised that could affect generalization to other cohorts; we, therefore, rated RoB as high. On the other hand, the work was transparent enough with possible reproducibility by releasing the code, hence LoTR was low. No specific concerns of privacy could be raised, thus we rated SPC low.

Beyond the scarcity of raw datasets, valuable information remains unexploited in complex scientific literature, particularly regarding the microbiome. Another study introduced DiMB-RE [70], a manually annotated corpus designed for mining diet-microbiome associations from scientific literature. The study assessed the performance of two systems, which included a fine-tuned BioBERT model [88] and LLMs, Generative Pre-Trained transformer (GPT)-4o models [89]. The results revealed that while GenAI models are capable of zero-shot extraction, their performance in detecting detailed entity relationships remains inferior to that of fine-tuned models. Moderate concerns of bias were identified due to the corpus’s restriction to biomedical literature and reliance on manual annotation introduce subjectivity and coverage constraints; we, therefore, rated RoB as moderate. The study reported core methodological elements of corpus construction and benchmarking sufficiently for reuse; hence, LoTR was low. No user or patient data were processed, as such, SPC was low.

To overcome extraction limitations and prevent hallucinations, recent efforts in PN emphasize the need for robust KGs to support GenAI models. Jackson et al. [71] introduced BASIL DB, a semantic KG that integrates bioactive compounds, food sources, and health outcomes extracted from clinical trials. Notably, the authors used natural language processing tools, including LLMs, during knowledge curation to automatically extract complex relationships between bioactive and phenotypic effects from unstructured biomedical text. The resulting resource reported coverage of 433 compounds, 7256 health effects, and 4197 food items coming from 40,296 abstracts. Evaluation on a subset of 100 relationships showed high precision in mapping bioactive-phenotype triples, though subset-only validation leaves unresolved the systematic extraction bias; therefore, we rated RoB as moderate. However, there was full transparency in reported outcomes and open-access code, leading to LoTR low, with no potential security concerns, hence, a low SPC.

With the establishment of robust data structures, the emphasis has shifted from modeling biology to semantic-aware dietary recommendations; however, while general recommendation systems are improving through standardized datasets, regional cuisines still lack such a coherent digital infrastructure. Gupta et al. [50] tackled this by proposing FKG.in, a specialized food KG for Indian cuisine, to address the lack of standardized digital nutritional data for regional recipes. The system utilized an MAS combining a nutrition data aggregator agent to combine information from multiple sources, and an LLM to normalize, translate, and harmonize unstructured recipe and ingredient data. Furthermore, a food composition analysis agent derived the total nutrient content and generated personalized dietary recommendations. The study demonstrated significant technical scalability by processing over 25,000 recipe instances, and was transparent in detailing how agentic workflows enabled the normalization of messy, culture-specific recipe data that traditional models could not process; therefore, LoTR was low. Reliance on LLM normalization across regionally variable web sources introduces a meaningful risk of silent nutrient-mapping errors that warrant validation against gold-standard assessments, leading to a rating of RoB as moderate. No risk of security and privacy concerns were identified since no handling of patient data, hence, we rated SPC as low.

#### 3.2.2. GenAI for Personalized Food-Effect Analysis

Helping individuals understand what a food contains, its outcomes, and whether it is appropriate for their health needs is a critical aspect for PN. GenAI systems increasingly augment this process by clarifying ingredient lists, highlighting potential risks, and interpreting product characteristics that are not immediately apparent from nutrient panels alone. Shekhawat et al. [72] contributed to this goal with the Food Label Analyzer, a system that combines Optical Character Recognition (OCR), Augmented Reality (AR), and a fine-tuned LLM to provide personalized, ingredient-level risk assessments based on a user’s health profile. The system extracted text from food label images using OCR, parsed the ingredient list, and applied a fine-tuned LLM, Phi [90], to evaluate each ingredient based on specific health conditions, such as diabetes, hypertension, pregnancy, and general health. For each ingredient, the model assigned a risk level (e.g., safe, low, moderate, or high), described associated health effects (e.g., “may increase blood sugar level”), and offered actionable recommendations. The system also integrated Google search to retrieve recent research articles and health news. All system outputs were displayed through an AR overlay directly on the food label image. The model was trained and evaluated on a custom dataset of food ingredients containing attributes such as category, health risk level, and condition-specific effects, achieving a micro-averaged accuracy of 91.4% on unseen ingredients using few-shot examples and beam search. Incomplete documentation of dataset construction and fine-tuning prompts limits reproducibility, giving an LoTR of moderate, and performance on a curated dataset may not reflect robustness in noisier real-world retail environments, with RoB being moderate. Additionally, security concerns were considered moderate, given the use of user information with an LLM that, to our knowledge, is open source and can be hosted internally, thereby reducing potential security and privacy risks, hence the moderate SPC rating.

A natural extension of ingredient-level interpretation is evaluating whether LLMs can provide accurate explanatory summaries of whole products, which is addressed by another work [73]. Here, authors evaluated the efficacy of LLMs, GPT-4 [91], in generating food product explanations. In the first part of the study, involving 12 registered dietitians, the researchers tested three levels of specificity: Level 1—basic product information, Level 2—nutritional facts and ingredients, and Level 3—individualized dietary needs. Evaluation across a five-point Likert scale in five food items showed that dietitians favored Level 2 and 3 over Level 1, with scores ranging from 3.7 to 4.5. However, experts noted that the outputs often contained misinformation, for instance, the model falsely recognized a product as gluten-free, or it suggested that gluten-free or lactose-free products are considered healthier, or lacked clinical alignment. This work clearly noted that it is key to involve experts within the process of validation of LLM output for nutrition counseling. To address these issues, the second part of the study developed a refined prototype integrating foundational knowledge and additional design changes in the instruction, though no quantitative re-evaluation was provided to confirm whether the refinements improved the initial metric scores. The prompt conditions and iterative refinement process were clearly documented, supporting reproducibility, so we defined LoTR as low, though the small food item set, subjective dietitian scoring, and the absence of independent evaluation of the refined prototype limit the strength of the conclusions, leading to a rating of RoB of high. Since no direct user information is used, even though the LLM used is provided through a third party, the security and privacy concerns were considered low; consequently, the SPC rating is low.

In a similar stream of thought, Yang et al. [74] proposed ChatDiet to further analyze the effect of food on different lifestyle variables, recognizing an inherent limitation of current LLM-based systems: the reliance on population-level nutritional knowledge. Therefore, they introduced a modular architecture that differentiates dietary knowledge from personalized causal reasoning. A personal model built over longitudinal dietary and health records applied causal discovery and inference to estimate individualized nutrient-outcome effects, such as the effect of specific nutrients on deep sleep duration, while a population model provided general Food Composition DB (FCDB)-related information. An orchestrator mediated between these two models, utilizing an LLM solely as a constrained synthesis layer to integrate outputs rather than generate recommendations independently. The evaluation involved 400 queries for 100 synthetic participants using an LLM, GPT-3.5 Turbo [92], across four target outcomes: heart rate variability, rapid eye movement sleep, overall sleep quality, and deep sleep duration. The model achieved a 92% average recommendation effectiveness ratio, defined as the proportion of recommendations consistent with the personalized causal model and health target. While results were promising, the evaluation relied entirely on synthetic profiles: a clear risk of bias, and hallucinations (e.g., recommending almonds despite a negative tryptophan effect) highlight that architectural feasibility does not yet translate into reliable real-world nutritional guidance, leading to RoB being high. Direct usage of a third-party LLM provider raised concerns on security and privacy, especially when handling user data—a concern not addressed in the work; accordingly, the SPC rating was classified as high. While the main approach and outcomes were reported, key implementation and evaluation details were incomplete, resulting in a moderate LoTR.

While the above studies focused on understanding a food’s properties, Yang et al. [57] examined a complementary dimension: whether foods deemed “appropriate” are actually feasible or suitable given a patient’s real-world constraints, specifically for individuals with cardio-metabolic conditions. They proposed an LLM-based multi-agent coaching workflow utilizing Gemini-1.5 Pro [93] that identified the practical barriers behind dietary lapses and delivered strategies tailored to those barriers. Following a user research study with 16 participants, which established a preference for a supportive rather than authoritative coaching style for the LLM, the authors defined 28 specific nutritional lapse barriers (e.g., geographic limitations, limited time, present bias). The MAS consisted of a barrier identification agent and a strategy execution agent. The system was validated using surveys from six real-world users and a simulation study involving 187 patient vignettes. Among the six real-world users, five found the assistant helpful, and four strongly agreed they learned something new. For the simulated data, two expert clinicians evaluated the outputs of a subset of 30 cases each, grading barrier identification accuracy at 91.5% and tactic comprehensiveness at 80%. When compared to a single-agent baseline, experts preferred the multi-agent workflow 70% of the time, a sentiment mirrored by an LLM, a GPT-4o [89] auto-evaluator, which showed a 66.7% preference for the proposed model when evaluated on a set of 153 high-quality vignettes. These findings indicated that multi-agent LLM systems gave insights into why eating habits break down and provided personalized, actionable solutions, offering a scalable way to strengthen long-term adherence to healthier diets. The work transparently shared the prompt template. On this basis, LoTR was low; however, evaluating only six users limits generalizability and risks bias, leading to an RoB rating of high. Furthermore, using ChatGPT as a judge, a cloud-hosted LLM, raises security concerns in case outputs of the previous LLM in the pipeline, i.e., Gemini, contained sensitive user information, giving an SPC of moderate.

#### 3.2.3. GenAI for Personalized Diet Recommendations

A critical component of personalized nutrition is delivering tailored recommendations that integrate a person’s metabolic state, nutritional needs, health goals, and real-world circumstances into concrete, individualized guidance. Agne & Gedrich [75] took a step back and focused on the safety aspect of recommendations to understand whether LLMs, ChatGPT [91], could provide comparable performance to an established structured guideline-based system to deliver tailored dietary advice based on an individual’s diet, phenotype, and genotype, namely the Food4Me algorithm [6]. The study utilized baseline data from 20 obese participants in the German Food4Me sub-cohort, inputting their anthropometric and dietary parameters. This study was among the few reviewed works that integrated complex biological markers, including blood values for glucose, cholesterol, and carotenoids, alongside genetic mutations. All these parameters were then input into ChatGPT using standardized prompts. The LLM outputs were compared with the official Food4Me feedback. Although ChatGPT sometimes aligned with Food4Me and provided clear explanations, it frequently showed inconsistencies, struggled with numerical inputs, gave unreliable macro- and micronutrient guidance, and was unable to link nutrient intake to specific foods. Its responses were only partly reproducible, and the quality of advice depended strongly on how precisely the user framed their questions. The work further highlighted that the provision of personalized recommendations by LLMs was promising and increased accessibility to personalized nutrition. However, laypersons should not rely on it without expert oversight due to potential misinformation risks. Generally, the work demonstrates documentation of methodology in depth; thus, we rated the LoTR as low. In this work, a small sample size could raise risks of bias; therefore, RoB was moderate, and the input of sensitive participant health data, including genetic and blood markers, into a third-party LLM raises privacy and security considerations that the study does not address, leading to an SPC of high.

Addressing the issue of hallucinations and errors in LLMs’ responses, as noted by previous authors, Liu et al. proposed KG-DietNet [76] whereby LLM output is grounded, through a RAG pipeline, on reliable nutrition data coming from the United States Department of Agriculture (USDA) FCDB [94] by building a KG. This was enhanced by a graph neural network, tasked to refine the embeddings in the KG to enhance nutrient relationships and user constraint representations while generating and scoring candidate foods based on user profiles, energy targets, and dietary restrictions. The LLM, GPT-4 [91] or GPT-3.5 Turbo [92], acted as a constrained synthesis layer. Their evaluation used 100 synthetic user profiles spanning 1500–3500 kcal targets with diverse preferences, allergies, and health constraints, emphasizing a wide range of dietary scenarios. The system outperformed other similar works [74,95], achieving a lowest mean absolute error of 65.1 kcal, alongside 10–15% improvement in nutrition alignment, as well as recorded the highest in user satisfaction in an ablation study comprising 50 participants, and they further put forth that these gains stemmed from the KG constraints rather than the LLM alone. This work successfully demonstrated how GenAI gave more sound suggestions when it uses validated data rather than generating from its own knowledge, though reproducibility depends on access to the pre-built KG and model hyperparameters. Accordingly, LoTR was moderate, and the reliance on synthetic profiles with a constrained ingredient set limits conclusions about real-world generalizability with potential risks of bias, RoB of moderate. The use of potential user profiles and constraints in a third-party hosted LLM-mediated pipeline without stated safeguards motivates SPC as moderate.

To reduce numeric and factual failures, Gavai & van Hillegersberg [77] illustrated how retrieval and rule-checking can be layered into generation to enforce guideline adherence. They presented a RAG-based recipe recommendation system that tries to move past the generic and often unreliable advice produced by a locally hosted LLM when it works as a standalone. They further demonstrated their proposed framework through one concrete example: generating smoothie recipes, which could potentially be expanded to recipes in the broader sense, for individuals managing obesity or Type 2 Diabetes (T2D). The system utilized RAG to integrate evidence-based information from national dietary guidelines with data from the FCDB, seasonal food produce lists, and curated diabetes-friendly recipes. Further, a virtual nutritionist dynamically refined recipes during generation to ensure predefined thresholds for glycemic control, calories, fiber, and fats are met. The authors evaluated system performance on 1000 generated recipes, reporting 80.1% adherence to nutritional guidelines, with most thresholds, except for fiber, being respected. Conversely, the system excelled in sustainability, achieving 92% compliance by prioritizing seasonal and locally sourced ingredients. Failure points were primarily driven by naturally high-sugar fruits and low-fiber ingredient combinations. The study concluded that while the RAG-based approach effectively bridges the gap between complex dietary advice and practical application, further optimization is required to strictly manage glycemic impact. Overall, the study demonstrated the potential of scalable, locally hosted LLM tools to deliver privacy-conscious, evidence-based nutrition that supports both individual health and environmental goals. However, the evaluation remained confined to proxy adherence metrics on a constrained recipe task, and the absence of real-user validation and physiological outcome testing limits conclusions to technical feasibility rather than clinical effectiveness; hence, RoB was moderate. The design minimizes external exposure, hence SPC was low, and the core approach and evaluation are described with sufficient detail to reproduce, resulting in a low LoTR rating.

Similarly, Benfenati et al. [78] used a RAG pipeline that grounds LLM responses in a consolidated, verified nutrigenetics DB, preventing the model from generating unsupported gene-diet claims for answering nutrigenetic questions. They further compared and assessed the performance of their proposed work coupled with two different LLMs, namely Mistral-7B [96] and GPT-3.5 Turbo [92]. The system was evaluated using 20 specific questions covering four key subdomains: food intolerances, food allergies, diet-induced oxidative stress, and xenobiotic metabolism. Expert reviewers assessed the model outputs across four metrics of relevance, depth, evidence-based support, and accuracy based on a 5-point Likert-type scale. Results demonstrated that the RAG implementation consistently improved performance for both models compared to their non-augmented baselines, particularly in accuracy and evidence-based support, where scores frequently rose from low ranges (1–2) to high ranges (4–5), with Mistral-7B + RAG demonstrating slightly higher performance. With their work, authors showed how RAG provided a computationally efficient and secure alternative to fine-tuning, ensuring that GenAI-generated dietary advice was grounded in the most recent scientific literature. The evaluation, however, rested on 20 questions assessed by a small, non-blinded panel of unidentified expertise, leaving the generalizability of these gains to broader nutrigenetic domains open, leading to a moderate RoB. The main approach is reported, but key evaluation details remain incomplete; as a result, LoTR was considered moderate. No user-specific personal data handling was required in this setup; consequently, SPC was considered low.

Alongside grounding and rule enforcement, other works study whether LLM meal plans can satisfy explicit quantitative constraints under standardized FCDBs. Focusing on whether LLM meal plans could meet strict quantitative energy goals when grounded in a verified FCDB, Khamesian et al. proposed NutriGen [79], which generated meal plans under user preferences and energy and nutritional constraints. The system built its FCDB on top of the USDA nutrient tables [94], incorporating user dietary history and preferences, and encoded this information into a structured prompt comprising the user profile and energy targets, task description, and output indicators to ground LLM-based meal plan generation. Evaluation involved multiple LLMs (Deepseek-V3 [97], Gemini 2.0 Flash Exp, Gemini 1.5 Pro [93], GPT-4o, GPT-4o Mini [89], GPT-3.5 Turbo [92], Llama 3.1 8B [98]) and used synthetic profiles constructed from 200 randomly selected USDA food items, each paired with an energy intervention goal between 1500–3500 kcal, generating three meal plans per LLM for a total of 30 outputs per model. Performance was quantified against ground-truth nutritional values, which, even though it showed promise with errors to energy targets as low as 1.55% with Llama 3.1 8B [98], it revealed substantial model-dependent variability. The fully documented prompt templates, code, and dataset construction on GitHub ensure transparency and reproducibility, thus LoTR was low. On the other hand, authors openly scoped the evaluation to a controlled synthetic benchmark as a first step, leading to a grade of moderate for RoB. However, they stated leaving real-world user studies and adaptive feedback for future work. Potential privacy concerns could arise in such a setup if real user data were used with a third-party hosted LLM, such as GPT-4o, a problem which was not recognized in the work; therefore, SPC was high.

Narrowing the purpose of LLM further down, in a parallel stream, Aydin et al. [80] approached the problem from an interface-centric perspective. Here, the LLM was not a decision-maker but a natural-language mediator whereby an LLM, Mistral 7B [96], interpreted textual dietary requests and further translated them into structured constraints that allowed filtering of the USDA’s FCDB [94] for further meal planning. Daily energy needs estimation was handled separately by a traditional machine learning model. Evaluation of the LLM component on 30 simulated user queries reported 91% accuracy in extracting structured constraints, though performance showed a decline for multi-intent requests like asking different types of diets into one query, such as “Mediterranean spicy low carb dinner” or due to ambiguity like “light dinner with protein”. The authors further ran a small usability pilot study with five participants, which demonstrated that users liked the interactive part of the system, though the very small pilot, the reliance on simulated queries, and the marginal improvement of the ML component over classical formulas collectively limit the strength of these claims; accordingly, RoB was high. While the main approach was reported, key implementation details were incomplete; thus, LoTR was moderate. The system showed usage of an LLM that can be locally hosted, without third-party processing, thus SPC was low.

Whereas the above systems emphasized constraint satisfaction and structured interfaces, another work moved on to a more user-facing approach by introducing NutriFlow [81], a diet recommendation system designed to generate personalized nutrition plans by leveraging user-specific data, including demographics, medical history, and dietary preferences (vegan or not). The system used an LLM, ChatGPT 3.5 Turbo [92], to analyze user inputs and produce precise dietary plans that match user needs, and the outputs were interfaced to the user through a chatbot. Beyond meal planning, the platform promoted holistic wellness through additional modules that monitor water consumption, suggest yoga exercises, and offer healthy recipes. Results from the study indicated that Nutriflow’s personalized suggestions led to user-reported improvements in health outcomes, specifically regarding cholesterol levels and blood pressure control. These claims, however, rest entirely on unverified self-report without controls, objective measurements, or a defined study population, thus RoB was high, and the collection of sensitive medical and demographic data through a third-party LLM API without described anonymization or retention controls raises additional concerns about deployment safety, leading to an SPC of high. Additionally, the limited methodological and evaluation detail prevents verification, thus LoTR was high.

Beyond text-only pipelines, another thematic line of work combined multimodal signals and conventional predictive models to support personalization from real-world inputs [82]. The authors presented NUTRIC AI, a system that delivered recommendations based on users’ clinical history and dietary habits. The system first collected users’ medical history and dietary information, then, using Convolutional Neural Networks (CNNs) and OCR, it extracted nutritional values from food images. Furthermore, by using a Long Short-Term Memory model, the system leveraged health history data to forecast future nutritional needs. Afterward, a recommendation system provided real-time recommendations that combined collaborative filtering to suggest user-preferred food with similar flavors and content-based filtering to suggest food based on personal nutritional requirements. To improve transparency, the system used Shapley Additive Explanations (SHAP) to provide explanations for each dietary recommendation, helping users understand the underlying decision process. For evaluation, the authors reported testing the system on 4000 user profiles, reporting 95% accuracy in meal plan recommendations and 92% user satisfaction. These figures, however, are difficult to interpret meaningfully, as the study provides no definition of how accuracy is measured, no dataset provenance, no evaluation protocol, and no model configurations; thus, LoTR was high with potential biases, with RoB high, rendering the reported performance unverifiable. Furthermore, since the collection and processing of sensitive clinical histories through the system raises serious privacy concerns, as no information on consent, anonymization, or data retention controls is described, a high SPC rating was attributed.

Hakim et al. [83] introduced NutriGuard, a multi-modal AI framework that generated personalized dietary recommendations by combining OCR, deep learning, and a fine-tuned LLM. The system used multilingual OCR and a CNN-based food classification model to process low-quality food images, along with manual input to capture nutritional information when needed. It incorporated a detailed user health profile that included biometric data, health conditions, and lifestyle factors. NutriGuard relied on LLM, Llama-3.2 (8B) [98], fine-tuned on clinical guidelines from the American Diabetes Association, the National Institutes of Health, and peer-reviewed nutrition studies, to assess food suitability and provided tailored outputs such as potential health benefits and risks, alternative food options, and dietary advice. For evaluation, the authors used a publicly available dataset of paired patient–doctor dialogues, achieving 85% validation accuracy. They conducted a qualitative review of 500 recommendations by certified nutritionists, reporting 87% clinical appropriateness. The system also correctly identified 91% of high-sodium foods relevant to hypertensive patients and 89% of high-glycemic foods for individuals with diabetes. Despite its effectiveness in personalized risk assessment, food substitution, and meal planning, the authors noted limitations, including occasional mismatches with dietary practices and reliance on static medical literature. While the implementation is well-documented, the evaluation remains constrained by a limited food product set and a nutritionist review that lacks independence; on this basis, RoB was moderate. The implementation is well-documented, thus LoTR was low. The system processed information of user health profiles through an LLM, which can be locally hosted; however, the concerns of privacy that might arise were not addressed, and SPC was moderate.

These design and grounding choices become particularly consequential in clinical settings, where personalization must align with disease-specific constraints and safety requirements. Addressing the critical need for expandable support systems in clinical oncology, research [84] evaluated how LLMs, including ChatGPT [91] and Gemini [93], can provide meal plans for breast cancer patients. The authors compared the quality of LLM-generated meal plans and grocery lists against those crafted by human professionals, focusing on the ability of GenAI to tailor advice. Thus, 31 prompting templates were used to evaluate recommendations based on the following variables: cancer stage, comorbidity, location, culture, age, dietary guideline, budget, and store. These Al-generated responses were compared against meal plans developed by four board-certified oncology dietitians to assess nutritional accuracy and personalization. Results indicated that while LLMs successfully adapted plans to location, cultural preferences, and budget constraints, they struggled to tailor recommendations for age, disease stage, or specific comorbidities. Gemini was distinguished by its ability to provide comprehensive details, including visual aids and specific pricing, compared to ChatGPT’s more text-heavy responses. Quantitatively, while dietitian-generated plans were more aligned with the USDA’s estimated daily calorie needs, the LLMs demonstrated better adherence. The study found no significant statistical difference between the overall quality of meal plans generated by the LLMs and the dietitians, though the small dietitian sample and single-run zero-shot prompts limit the robustness of this equivalence finding. The models also displayed a bias toward American cuisine, which the authors acknowledged as a limitation that partially undermines the broader health equity argument. Given the above two statements, the RoB was moderate. The study nonetheless concluded that LLMs represent a scalable and accessible tool for improving health equity in oncology nutrition, particularly for patients facing socioeconomic barriers, with professional oversight remaining essential to ensure accuracy and patient safety. Since the evaluation design and conditions are clearly reported, LoTR was low, and as the study uses scenarios rather than identifiable patient records, SPC was low.

In diabetes care, Lafqih et al. [85] evaluated the performance of two chatbots using two different LLMs, namely GPT-4 [91] and Gemini 1.5 Flash [93], in delivering nutritional recommendations. Chatbots’ response performance was evaluated using 52 simulated patients spanning nine diabetes-related categories, ranging from uncomplicated diabetes to complex scenarios involving comorbidities like chronic kidney disease (CKD) and malnutrition. Patient case information comprising demographics, biological markers coming from laboratory test results, comorbidities, and medications were further supplied to the chatbots. The chatbots were further evaluated across criteria such as clinical relevance, alignment with guidelines, personalization, and practicality. Quantitatively, both models closely matched the expert’s daily caloric targets (2172 Kcal), though Gemini recommended a significantly higher protein ratio (21%) compared to ChatGPT, while the latter recommended a higher carbohydrate ratio (50%) and no differences in terms of fat recommendations. Results overall also showed that Gemini significantly outperformed ChatGPT in overall guideline concordance, particularly in complex clinical scenarios. Conversely, no significant performance discrepancies were observed between the models in categories such as diabetes with cardiovascular complications or eating disorders. Despite Gemini’s superior scoring, a qualitative review noted that both models occasionally provided impractical food suggestions, though Gemini demonstrated better safety awareness by frequently advising users to consult Healthcare Professionals (HCPs). The study highlighted that model selection can affect compliance with clinical standards and that professional oversight remains essential to correct inaccuracies. The conclusions were well-supported by a systematic and transparent evaluation design, hence having a low LoTR. On the other hand, RoB was moderate, given the fact that only the most clinically relevant of the three outputs per model was retained and further evaluated by an expert without acknowledging the worst-case scenario implications. Usage of a third-party-hosted LLM, Gemini, warrants further security measures in case real patient data was to be used, which was not acknowledged. This is an SPC rating of M, given that no real user data was used.

Onay et al. [86] then provided a complementary “LLM vs dietitian” test that separates qualitative safety (avoiding contraindicated foods) from quantitative correctness (hitting nutrient targets). They evaluated GPT-4’s [91] ability to generate dietary plans for a simulated patient with obesity and six chronic disease scenarios: obesity + T2D, celiac disease, lactose intolerance, hypercholesterolaemia, hypertension, and CKD across 18 unique prompts (i.e., six diseases x three repetitions). The outputs were assessed using three criteria: accuracy (adherence to recommended nutrient ranges, e.g., 45–55% energy intake from carbohydrates for people with diabetes), reliability (exclusion of contraindicated food items, e.g., high-potassium foods for people with CKD), and attractiveness (food variety, based on the diversity and repetition of food items). While the LLM achieved perfect reliability by consistently avoiding restricted foods, it demonstrated moderate attractiveness (scoring 4 out of 6) and failed to meet clinical micronutrient and energy requirements in all six scenarios. The generalizability of these findings was limited by the use of a single hypothetical patient profile and a single dietitian for comparison, and the model was evaluated using basic prompts without iterative refinement or grounding in a domain-specific knowledge base, leading to a high RoB. Despite these constraints, the evaluation criteria were predefined and clearly operationalized, and full prompts, outputs, and scoring rubrics are publicly available, fully supporting reproducibility and independent verification of the reported findings, leading to a low LoTR. As the study is based on a simulated patient, SPC was moderate, as concerns might arise if real patient data is used with the third-party-hosted LLM.

Finally, beyond clinical and methodological constraints, the linguistic and regional context itself limited personalization quality even when the same model is used. In another study, which focused on Central Asian populations, the authors [87] evaluated an LLM’s, GPT-4 [91], ability to provide personalized nutrition advice in English, Kazakh, and Russian. They generated a dataset of 50 patient profiles comprising demographic, anthropometric, biomarker, and lifestyle information, which was validated by medical professionals and translated from Kazakh to Russian and English. GPT-4 received each profile along with two different prompts requesting dietary recommendations and a diet plan for the day, incorporating Central Asian foods. The responses were evaluated by three evaluators using a 5-point Likert scale for personalization, consistency, practicality, and availability. While performance in English and Russian was moderate across all criteria (mean scores between 3 and 3.5), the model performed poorly when handling Kazakh inputs, with average scores close to 1 and frequent hallucinations or incoherent suggestions. The work clearly outlined the methodology, giving a low LoTR. On the other hand, given the fact that ratings like Likert are subjective and that experts assessed translations rather than native LLM responses, leads to an RoB rating of moderate. As the study is based on a simulated patient, SPC was moderate, as concerns might arise if real patient data is used with the third-party hosted LLM.

## 4. Discussion

Across the reviewed literature, GenAI-enabled PN showed a consistent movement toward stronger grounding through structured resources such as KGs, curated corpora, and retrieval-augmented pipelines. However, it is critical to distinguish between different levels of grounding quality, as the efficacy of these architectures is fundamentally tied to data provenance and curation methodology. While systems backed by rigorously curated, expert-validated DBs offer highly reliable guardrails, a recurring vulnerability in the reviewed literature is that many semantic foundations were constructed or expanded using automated extraction methods and were subsequently treated as inherently trustworthy. This was evident in KG pipelines, where LLM-assisted extraction accelerated curation, but this further risks silently embedding incorrect relations without systematic validation [71]. As a result, downstream models may inherit flawed information while still trying to produce logical rationales, reinforcing that grounding alone does not guarantee correctness unless the underlying structure is verified, versioned, and uncertainty-aware [71]. This lack of rigorous data verification is compounded by a significant lack of standardization in how nutritional knowledge is represented. Without common ontologies or standardized data schemas, the “grounding” remains siloed, hindering the development of universal benchmarks for model accuracy. A similar reliability concern appeared when LLMs were used to normalize and fill gaps in regional food composition resources. Such unverified LLM-generated estimates and nutrient mappings can weaken confidence in the derived nutrition outputs in the absence of quantitative validation or real-world error analysis [50].

A critical hurdle identified across these systems was the lack of interoperability. None of the reviewed works addressed the issue of seamlessly exchanging data with electronic health records, wearable devices, or diverse laboratory DBs [99]. While interoperability should not be framed as a universal definitional prerequisite for all conceivable forms of PN, particularly approaches grounded in static, genotype-based stratification, it is important to distinguish between minimal data acquisition requirements and broader system-level integration. In contemporary GenAI PN paradigms, at least a basic level of interoperability with personal data streams such as wearable devices and sensors is functionally indispensable, as these inputs enable continuous monitoring and individualized model updates [6]. In contrast, large-scale integration with EHRs or institutional laboratory databases is not strictly required to classify an intervention as PN, but it remains a powerful facilitator for advanced, longitudinal personalization. Consequently, we view extended interoperability as a long-term infrastructure goal that enables GenAI-based PN systems to scale, reduce data fragmentation, and support dynamic, adaptive user profiles over time [8,99]. To move beyond siloed architectures, future research must focus on the native integration of GenAI within the existing healthcare infrastructure, which requires the adoption of universal nutritional data schemas and ontologies. While some ontologies exist [100], for instance, for establishing food classifications, research in standardizing these data schemas and frameworks remains limited. The current reviewed literature predominantly relies on study-specific, heterogeneous structures that lack a common framework. Significant work remains to standardize how nutritional knowledge is modeled, ensuring that internal AI representations align with global clinical taxonomies.

Furthermore, these semantic foundations need to be decoupled from the data exchange layer. Standardized data exchange protocols, such as the Health Level Seven Fast Healthcare Interoperability Resources framework [101], could help make systems more interoperable. With these foundations in place, GenAI can hence operate as an active node within the bigger healthcare ecosystem, enabling data flow between, for example, consumer wearables and extensive clinical records rather than functioning as an isolated tool, ultimately creating a more connected and genuinely holistic system. A related limitation was the reliance on indirect or simplified health metrics and incomplete biochemical specificity within large dietary datasets. Health-aligned recipe corpora can potentially increase guideline coherence but depend heavily on scores like Nutri-Score, which compress complex nutrient profiles into single aggregated values and thereby restrict clinical fidelity.

Web-scraped recipes further introduced cultural and culinary biases that do not reflect global dietary diversity, raising concerns about equity and generalizability when such datasets inform generative recommendation systems [102]. Even though structured nutrient DBs offered numerical precision in the reviewed works, they remained narrow in their coverage of mixed dishes, cooking methods, and region-specific ingredients [50,79,80]. This was eventually reflected in many grounded frameworks, which relied on culturally constrained depictions of food. This limitation was reinforced in image-grounded assessment pipelines that still inherited the DBs coverage bias and model-version bias, particularly for visually complex mixed dishes and non-represented cuisines [84]. In multilingual and culturally diverse contexts, the consequences of these limitations become more pronounced. Performance declined sharply in lower-resource languages, with higher hallucination rates and incoherent recommendations, underscoring that LLM capabilities do not transfer uniformly across global populations [87]. To actively reduce cultural bias and ensure health equity, future research must prioritize diversification through the expansion of nutrition-related datasets and retrieval corpora to comprehensively include beyond Western cuisines and dietary patterns, as well as optimizing models for languages with fewer resources.

Efforts in microbiome and nutrigenetics similarly highlighted the limits of current GenAI infrastructures. While annotated corpora supported improved relation extraction, they did not address the deeper scientific instability of domains characterized by contradictory findings and heterogeneous study populations [70]. Likewise, retrieval-augmented nutrigenetics systems mitigated hallucinations but remained tightly bound to the quality, completeness, and internal agreement of the underlying retrieval corpus, especially when variant evidence is weak or conflicting. In both areas, the unmet challenge extended beyond extraction accuracy to representing disagreement, weighting evidence quality, and producing calibrated recommendations under uncertainty [78]. Moreover, synthetic augmentation strategies proposed to address data scarcity may reinforce cohort bias when the original sample is small or unrepresentative, limiting biological validity despite improved predictive performance [69].

Within personalized nutrition recommendation systems, hybrid architectures increasingly constrain LLM behavior using causal models, KGs, or structured DBs. These designs improved control and reduced hallucinations. However, their performance evaluations often relied on synthetic profiles, simulated queries, or small usability cohorts, which further limited confidence in real-world robustness [74,76,79,80]. This widespread reliance on simulated environments and small sample sizes directly contributed to the High RoB ratings assigned to a significant portion of the reviewed literature, as these methodological compromises inflate perceived efficacy while masking potential clinical failures. Several systems also assumed the availability of dense longitudinal single-subject clinical trial datasets, which remain unrealistic for population-scale deployment [74]. This brings to light the issue of scalability; while a system may perform well for a handful of curated profiles, the computational cost and data-cleansing requirements for scaling these high-touch architectures to millions of diverse users remain largely unaddressed. Even when numerical alignment is strong, performance varies markedly across LLM backends, implying that reproducibility hinges more on model selection and prompt design than on stable system properties [76,79]. Related concerns about evaluation validity recur in systems that reported improvements without sufficient methodological detail (e.g., sample size, controls, duration) or surface-level scoring (e.g., Likert practicality ratings) and reliance on self-reported adherence. This can inflate apparent impact in the absence of objective monitoring [81]. Similarly, studies that prioritized guideline alignment using simulated clinical vignettes provide limited evidence of safety and effectiveness under real patient adherence behavior and live clinical constraints [85]. Comparative simulations against dietitians further suggested that high reliability in avoiding contraindicated foods does not imply nutrient adequacy or personalization, while the use of single hypothetical profiles and single comparators limited deductions on generalizability [86].

Beyond data constraints, most current systems remained fundamentally static. While they successfully generated coherent plans or educational content based on fixed user profiles and lifestyle habits, they lacked the capacity to adapt dynamically to real-time phenotypic signals, changing metabolic states, or evolving clinical contexts—requirements central to mechanistic and biologically driven PN [76,78]. To achieve holistic precision, future research must shift focus toward architectures that adapt through time by ingesting evolving user needs and preferences along with real-time physiological signals. Incorporating continuous metabolic telemetry, such as continuous glucose monitors or wearable biosensors, will allow these systems to dynamically adjust interventions based on immediate bodily responses and metabolic fluctuations, rather than relying on historical user clinical snapshots or static user preferences. Rule-based compliance in RAG recipe systems demonstrated this gap clearly: outputs satisfied fixed thresholds but did not incorporate physiological responses or long-term adaptation [77]. Similar limitations emerge in guideline comparison studies, where general-purpose LLMs imitated expert tone but failed in numeric consistency, nutrient-food mapping, and reproducibility over time [75]. Even multi-agent coaching systems that reported strong barrier-identification accuracy remained constrained by very small real-user samples, reliance on simulated vignettes, and dependence on predefined behavioral taxonomies that may not generalize to fluid real-world barriers [57].

Assessment-oriented systems faced related challenges. Real-world robustness of systems incorporating images is limited by visual ambiguity, lighting conditions, and camera variability [82]. Studies on ingredient decomposition showed high detection performance but weaker decomposition into atomic components, especially for oils, seasonings, and complex foods. Similarly, LLM-based explanation tools showed potential but often relied on small evaluation sets, leaving reproducibility unproven [73]. This generalizability concern also applied to OCR-driven label assessment pipelines, where validation on curated ingredient datasets and dependence on external web search for missing knowledge could limit reliability under noisy, real-world labels and incomplete domain coverage [72]. More broadly, stacked multi-module architectures that report very high accuracy and satisfaction on internal benchmarks require external validation on real food images, diverse diets, and clinically supervised outcomes; otherwise, robustness remains uncertain despite explainability add-ons such as SHAP [82]. Finally, multimodal clinical systems may achieve strong internal metrics yet still show cultural mismatch (e.g., portion norms), narrow food-category coverage, and limited adaptability to emerging evidence if grounded primarily in static guideline corpora [83].

Safety vulnerabilities could also arise when models encounter complex clinical scenarios, as shown by failures to incorporate texture modifications or drug–nutrient interactions in oncology nutrition planning [84]. Importantly, this should be interpreted in a risk-stratified manner: the evidence most strongly supports unsuitability for direct, autonomous integration into high-stakes clinical decision-making workflows, where errors in quantitative precision, contraindication handling, or disease-specific adaptation can directly affect patient safety. These patterns clearly demonstrate that current GenAI systems are not yet tailored for independent clinical usage without strict human supervision, and, particularly in this case, in order to move forward, GenAI systems require strict human-in-the-loop, specifically experts, oversight, and domain-specific safety modules before any consideration for clinical deployment [75,84]. Accordingly, the key limitation is not that all clinical-facing use is uniformly inappropriate, but that current systems lack the reliability, calibration, and validation required for unsupervised deployment in higher-risk clinical roles. By contrast, lower-risk applications, for instance in patient education [80], adherence coaching [103,104], draft meal-plan generation for clinician review [84,86], or structured information support [105], may still be useful as supervised adjuncts, provided their outputs are constrained, auditable, and reviewed by qualified professionals.

Two further cross-cutting issues of opacity and privacy remain largely unaddressed. Most systems depended on black-box LLM reasoning for extraction, constraint parsing, or recommendation synthesis, offering limited transparency into why decisions are made, especially when upstream data may be uncertain or biased [71,75,83]. This systemic opacity is directly reflected in our quality assessment, where several studies received a moderate or high rating for LoTR due to missing implementation details, proprietary model dependencies, or unavailable codebases. Furthermore, while we did not employ a formal, standardized privacy assessment tool during our methodology, we graded the SPC as shown in Table 2, which revealed a recurring vulnerability. Consequently, numerous applications were flagged with moderate or high SPC. Several pipelines required highly sensitive information (biomarkers, dietary histories, clinical conditions, or even genotype data) yet transmitted these data to cloud-hosted models in ways that are not always described in sufficient technical detail to assess privacy risk [87]. No reviewed system discussed or provided robust strategies for data governance. This systemic absence becomes critical as PN intersects with the handling of patient data and clinical regulation [78,83]. These concerns become especially acute in systems that explicitly depend on non-local hosted LLMs for clinical recommendation generation or evaluation, where patient data may be externally transmitted and processed without explicit protections or governance guarantees [75,84,85,87].

While past PN research showed evidence that meaningful personalization requires anchoring dietary and lifestyle profiles to objective biomarkers [106,107], our review reveals a profound gap in terms of leveraging biological and metabolic granularity, highlighting that GenAI applications in PN function at a level of partial personalization. Most reviewed works that put forth personalization focused narrowly on subjective contextual data such as preferences, allergies, macronutrient targets, or basic anthropometric data and did not operationalize biological determinants as active, mechanistic, and uncertainty-aware drivers. A small subset of studies did engage biological content or biologically relevant inputs, including microbiome-related knowledge extraction [70], nutrigenetics retrieval-grounded question answering [78], and biomarker-containing patient profiles used in recommendation evaluation in multilingual settings [87] and genotype- plus biomarker-informed dietary recommendation generation [75]. These studies indicate that biological information is not absent from the reviewed literature and, when included, can meaningfully contribute to personalization relative to systems that rely only on preferences or basic demographic/contextual data. However, in most cases, these biological variables were incorporated as profile-level inputs to prompting or rule contexts rather than as explicitly modeled, evidence-weighted, uncertainty-aware drivers of mechanistic personalization that can adapt over time. The latter provided the clearest example of biologically informed personalization within a structured PN-style comparison workflow, although the LLM showed substantial reliability and numerical limitations [75]. Consequently, current GenAI approaches remain closer to the preference-aware aspect rather than to the biologically grounded frameworks, limiting their ability to translate complex biological heterogeneity into safe and actionable dietary guidance.

A promising way to address this gap is to integrate causal machine learning frameworks with GenAI systems [57], enabling individualized effect estimation and counterfactual reasoning [9] that move beyond purely associative pattern recognition toward mechanistically grounded personalization. By explicitly modeling cause–effect relationships between dietary exposures, biomarkers, and outcomes, such hybrid architectures could support biologically grounded, effect-aware personalization rather than correlation-driven recommendation generation.

Moreover, it is important for us to highlight the limitations of our review process. The search strategy relied on three major DBs and English-language keywords, which may have excluded relevant studies indexed exclusively in other scientific repositories or reported in non-English terminology. Additionally, variations in indexing practices across platforms meant that some works were not captured by multiple DBs, thereby introducing the possibility of missed eligible studies despite systematic screening procedures. Methodologically, due to the substantial heterogeneity and proof-of-concept nature of current GenAI applications, established methodological AI quality assessment tools could not be strictly applied. We adapted core principles from these frameworks to evaluate methodologically LoTR, RoB, and potential SPC. We raised concerns about security and privacy issues while noting that our critiques are based on our evaluation, as per criteria in Table 1, of the reported system architectures rather than a dedicated security audit.

Before real-world or clinical deployment, these systems must be built on proven data while undergoing strict testing across various medical settings. They also require transparent reasoning workflows that HCPs can audit and governance protocols to protect privacy, e.g., keeping sensitive data on local protected servers rather than in the cloud. Future work must focus on establishing standardized protocols for data exchange and model evaluation to ensure that these systems are not only accurate but also scalable and interoperable across the global ecosystem. They must also demonstrate closed-loop adaptivity to real-time physiological signals rather than relying on static user profiles. Critically, GenAI systems must operate within human-in-the-loop frameworks where HCPs can supervise, override, and contextualize recommendations. Multiple works to date [43,44,45,46,75,86] showed that LLMs are unable to deliver the mathematical precision, which can affect safety. Instead, LLMs could possibly function in larger MAS that delegate quantitative tasks to validated tools such as automatic dietary assessment systems or allergy prediction models [16,17,18,53,54,55,56,57,58]. Without addressing all of the above limitations and implementing safeguards, the current generation of GenAI nutrition systems, despite promising progress, remains unsuitable for direct, autonomous clinical integration in high-stakes decision-making contexts. However, selected lower-risk, supervised uses may be feasible as adjunctive support tools. We further summarized the limitations highlighted during the review and put forth an actionable blueprint for the way forward for GenAI applications in PN in Table 3.

## 5. Conclusions

This review of 21 original works has shown that current GenAI applications in PN are dominated by LLMs, with the largest body of work focusing on the generation of personalized dietary recommendations, for instance, meal planning. Personalization, as works put forth, is mostly achieved through user contextual data like preferences and certain lifestyle variables rather than through biomarkers or mechanistic determinants of diet response. Accordingly, microbiome-, genomic-, and broader multi-omics integration remains limited to truly fulfill the PN aspect.

We note that the evidence base is moreover constrained by questionable evaluation practices with multiple assessments of synthetic data, which confound verdicts on performance, robustness, and generalizability in real-world settings, driving the moderate–high RoB ratings observed across the field. Consequently, a persistent theme among limitations was hallucinations, inconsistent numeric reasoning, and poor mathematical precision, which clearly impede deployment in high-stakes settings without strong supervision. These limitations are further heightened by gaps in data verification, interoperability, and standardization, whereby inconsistent knowledge representations and consensus ontologies or schemas hinder comparability across systems and slow progress toward shared evaluation benchmarks and safety practices, reflected in the moderate LoTR ratings. In parallel, several pipelines require sensitive personal health information, yet provide insufficient detail to assess security and privacy issues, particularly when cloud-hosted models are used, as shown by the moderate to high SPC ratings assigned in this review.

Taken together, the literature suggests that near-term progress toward holistic PN will depend less on using LLMs as stand-alone decision makers and more on embedding them within broader, safety-oriented frameworks. One such example could be the inclusion in MAS of over-validated data sources, explicit rule or constraint checking, and tool-based computation for quantitative tasks. Crucially, to ensure patient safety, expert validation should be treated as a core requirement rather than a supplementary step, especially where GenAI outputs may influence higher-risk clinical decisions; in lower-risk supportive roles, these systems may still have value as supervised adjuncts within clearly bounded use cases. Substantial room for improvement remains before GenAI can deliver clinically reliable, biologically grounded, and culturally equitable PN. This includes the development of diversified nutrition-related datasets and corpora towards more inclusive GenAI systems, stronger real-world validation, robust hallucination mitigation, and deeper integration of biological signals to move beyond user-context personalization. Ultimately, bridging these gaps, while ensuring expert oversight, is essential not just for technical improvement but for unlocking the potential of GenAI in reducing the global burden of diet-related chronic disease through personalized care.

## Figures and Tables

**Table 1:** Assessment criteria used to rate Lack of Transparency and Reproducibility (LoTR), Risk of Bias (RoB), and Security and Privacy Concern (SPC).

Assessment Criteria	Low	Moderate	High
LoTR	Study clearly reported core methodological elements with sufficient detail to understand and reasonably reproduce the setup.	Main approach and outcomes were reported, but key implementation or evaluation details were incomplete.	Indicated insufficient methodological detail to verify and/or reproduce the reported claims.
RoB	Limited concerns in the data source, methodology, and/or evaluation design.	Identifiable concerns (e.g., small samples, simulated cases, subjective scoring, or narrow scope), but still interpretable findings.	Substantial concerns likely to weaken the validity or inflate performance estimates.
SPC	Minimal privacy or security concerns; avoids unnecessary exposure, minimizes external dependencies, and specifies safeguards.	Some concerns; includes external components or incomplete controls. Risks are contained by mitigation strategies.	Substantial concerns; relies entirely on external components processing for user-specific contexts without mitigations mentioned.

**Table 2:** Summary of the selected studies: GenAI methodologies and PN applications. DT: Data Type. VS: Validation Source. LoTR: Lack of Transparency and Reproducibility, RoB: Risk of Bias, SPC: Security and Privacy Concerns, L: Low, M: Moderate, H: High.

Study	Keywords	DT/VS	Summary	LoTR	RoB	SPC
Knowledge and Data Foundations						
Xiangyu and Hao (2025) [69]	“GAN”, “generative adversarial network”/ “personalized nutrition”	Real-world + Synthetic/ Automated	Used a Wasserstein GAN with Gradient Penalty (WGAN-GP) to augment small datasets with realistic synthetic performance and supplementation-response samples, improving prediction accuracy for carbohydrate-protein recommendations in data-scarce endurance settings.	L	H	L
Hong et al. (2025) [70]	“large language model”/ “personalized nutrition”	Real-world/ Automated	Introduced a manually annotated corpus for diet–microbiome association mining and benchmarked fine-tuned BioBERT against large language model (LLM)-based zero-shot extraction, finding fine-tuned models outperformed LLMs for detailed relation mapping.	L	M	L
Jackson et al. (2025) [71]	“LLM”, “large language model”/ “personalized nutrition”	Real-world + Synthetic/ Expert	Built a semantic Knowledge Graph (KG) integrating bioactives, food sources, and health outcomes extracted from biomedical literature using natural language processing tools, including LLMs; reported coverage of 433 compounds to support grounded reasoning.	L	M	L
Gupta et al. (2024) [50]	“LLM”, “personalized nutrition”	Real-world/ Automated	Proposed an Indian cuisine food KG and agentic pipeline using a nutrition aggregation agent plus an LLM to normalize and harmonize unstructured recipes; scaled to over 25,000 recipes to enable coherent composition analysis and downstream recommendations.	L	M	L
Food-Effect Analysis						
Shekhawat et al. (2025) [72]	“LLM”, “large language model”/“personalized nutrition”	Real-world/ Automated	Combined Optical Character Recognition (OCR), Augmented Reality (AR), and a fine-tuned LLM to extract label text, interpret ingredient-level risks for user conditions (e.g., diabetes, hypertension, pregnancy), and presented actionable guidance via an AR overlay; reported strong ingredient-level accuracy on a custom dataset.	M	M	M
Szymanski et al. (2024) [73]	“LLM”, “large language model”/“personalized nutrition”	Real-world/Expert	Evaluated Generative Pre-trained Transformer (GPT)-4-generated food product explanations under increasing prompt specificity and found higher perceived usefulness for nutrition- and user-specific prompts, but noted misinformation and clinical misalignment, motivating expert-in-the-loop prompt/template refinements.	L	H	L
Yang et al. (2024) [74]	“LLM”, “large language model”/“personalized nutrition”	Real-world + Synthetic/ Automated	Separated population-level food composition knowledge from individualized causal modeling over longitudinal records; used an LLM as a constrained synthesis layer to analyze the effects of nutrition on physical health indicators, which can be further used for targeted recommendations.	M	H	H
Yang et al. (2025) [57]	“LLM”, “large language model”/ “personalized nutrition”	Real-world + Synthetic/ Automated + Expert + User	Multi-agent LLM coaching identified barrier types behind dietary lapses and delivered tailored behavior-change tactics; validated in user studies and clinician review with strong barrier identification accuracy and expert preference over a single-agent baseline.	L	H	M
Diet Recommendations						
Agne & Gedrich (2024) [75]	“ChatGPT”/“personalized nutrition”	Real-world/ Automated, Human annotator	Compared ChatGPT with the structured Food4Me algorithm using obese participant profiles; found occasional alignment but frequent inconsistencies, weak numerical handling, and variable reproducibility, indicating risks without expert oversight.	L	M	H
Liu et al. (2025) [76]	“large language model”/“personalized nutrition”	Real-world + Synthetic/ Automated + User	Grounded LLM synthesis in a KG built from the United States Department of Agriculture (USDA) Food Composition Database (FCDB) within a Retrieval-Augmented Generation (RAG) pipeline, refining representations with graph learning; reports improved nutrition alignment and reduced calorie estimation error versus LLM-centric baselines.	M	M	M
Gavai & van Hillegersberg (2025) [77]	“RAG”/ “personalized nutrition”, “precision nutrition”	Real-world + Synthetic/ Automated	Used retrieval and rule-checking to generate guideline-aware recipes with a locally hosted LLM; integrated dietary guidelines and FCDB sources and reports 80.1% adherence across generated recipes while identifying typical failure modes (e.g., high-sugar fruits).	L	M	L
Benfenati et al. (2025) [78]	“RAG”, “large language model”/ “personalized nutrition”, ”nutrigenetic”	Real-world/ Expert	Employed a RAG pipeline grounded in a verified nutrigenetics knowledge base to answer gene-diet questions; expert evaluation shows improved accuracy and evidence support for both smaller and proprietary LLMs compared with non-augmented baselines.	M	M	L
Diet Recommendations						
Khamesian et al. (2025) [79]	“LLM”, “large language model”/ “personalized nutrition”	Synthetic/ Automated	Generated meal plans under explicit energy and nutrition constraints using a verified USDA-based FCDB and structured prompting; evaluation across multiple LLMs reports low energy-target error for top-performing models but substantial model-dependent variability.	L	M	H
Aydin et al. (2025) [80]	“LLM”/ “personalized nutrition”	Real-world/ Automated + User	Used a classic machine learning model for energy estimation and an LLM as a natural-language mediator to convert user requests into structured constraints for FCDB filtering; reports high constraint-extraction accuracy but reduced performance on multi-intent or ambiguous queries.	M	H	L
Dhote et al. (2024) [81]	“ChatGPT”/ “personalized nutrition”	Real-world/ Automated	User-facing chatbot system that generated personalized diet plans from user demographics and health inputs and includes lifestyle modules (e.g., hydration, exercise); reports improved health outcomes but provides limited quantitative substantiation.	H	H	H
Harish et al. (2025) [82]	“chatbot”/ “personalized nutrition”	Real World/ Automated + User	Multimodal pipeline combining food-image analysis (CNN/OCR), health-history forecasting, and hybrid recommendation methods; added Shapley additive explanations and reported high recommendation accuracy and strong user satisfaction.	H	H	H
Hakim et al. (2025) [83]	“LLM”/ “personalized nutrition”	Real-world/ Expert + User	Multimodal system combining OCR, deep-learning food classification, and a guideline-tuned LLM to assess suitability, propose substitutions, and provide recommendations; reported high clinical appropriateness and accurate disease-relevant food detection.	L	M	M
Logan et al. (2025) [84]	“LLM”, “large language model”, “ChatGPT”/ “personalized nutrition”	Synthetic/ Automated + Expert	Compared LLM-generated cancer meal plans with oncology dietitians; found good adaptation to culture/budget/location but weaker tailoring for disease stage and comorbidities. Highlighted that expert oversight remains essential.	L	M	L
Lafqih et al. (2025) [85]	“chatbot”, “ChatGPT”/ “personalized nutrition”	Synthetic/Expert	Benchmarked GPT-4 and Gemini chatbots across simulated diabetes scenarios; Gemini shows higher guideline concordance, especially in complex cases. Both models sometimes provide impractical suggestions, reinforcing the need for clinical oversight.	L	M	M
Onay et al. (2025) [86]	“ChatGPT”/ “personalized nutrition”	Synthetic/Human annotator	GPT-4 avoided contraindicated foods consistently but failed quantitative nutrient and calorie targets in all chronic-disease scenarios; demonstrated safe exclusions but poor quantitative precision without grounding.	L	H	M
Adilmetova et al. (2025) [87]	“ChatGPT”, “large language model”/ “personalized nutrition”	Synthetic/Human annotator	Evaluated GPT-4 recommendations across English, Russian, and Kazakh. Performance was moderate in English/Russian but very poor in Kazakh, with hallucinations and impractical outputs, showing the need for localized multilingual models.	L	M	M

**Table 3:** Failure modes and mitigation strategies across GenAI applications in PN.

Failure Mode	Risk/Impact	Mitigation Strategy
Level: Data Grounding & Curation—What is the input of the model?		
Unverified Knowledge Extraction	Hallucinations or incorrect relations embedded in generated corpora or KGs.	Include provenance metadata and confidence scores for extracted relations; apply human-in-the-loop verification.
Reductionism	Loss of nutritional fidelity due to oversimplified representations.	Use granular nutritional profiling; use high-dimensional data.
Standardization	Inconsistent schemas hinder reuse, comparison, and reproducibility.	Use expert-accepted schemas; publish relation definitions and mapping rules; enforce conformance checks.
Level: Data Grounding & Curation—What is the input of the model?		
Data Bias	Reduced equity and generalizability across diets, regions, and languages.	Broaden coverage of mixed dishes and region-specific foods; perform stratified evaluation by cuisine, language, and context; document coverage limitations.
Scientific Uncertainty	Overconfident recommendations despite conflicting or weak evidence.	Apply uncertainty-aware mechanism; surface disagreements; use retrieval credibility metrics (study type, peer-review status, recency, citations).
Level: System and Architecture—Why do models fail?		
Computational Imprecision	Numeric inaccuracies undermining dietary constraints and safety guarantees.	Delegate quantitative tasks to validated calculators or rule-based components using MAS architectures.
Data Privacy	Exposure or misuse of sensitive genetic and clinical information.	Prefer models hosted locally; apply encryption; define explicit data-access and processing boundaries.
Interoperability	Fragmentation of data prevents holistic and longitudinal PN profiling.	Define standardized PN profile schemas and interfaces; explicitly declare required fields and supported data sources.
Opacity Risks	Limited auditability of recommendation logic and evidence use.	Provide audit logs (retrieved evidence, applied constraints, rule checks); expose intermediate structured representations.
Static Processing	Failure to adapt recommendations to dynamic physiological or metabolic states.	Treat static assumptions as limitations; evaluate iterative update cycles using real measurements and observed outcomes.
Level: Clinical & User Utilization—What happens in the real world?		
Evaluation Validity	Inflated performance estimates with unclear real-world safety and effectiveness.	Conduct longitudinal studies involving real patient cohorts.
Superficial Personalization	Systems function as preference engines rather than biologically grounded precision tools.	Integrate multi-omic signatures as evidence-weighted drivers of recommendations.

## Data Availability

Data sharing is not applicable to this article, as no new data were created.

## Abbreviations

The following abbreviations are used in this manuscript:

AI	Artificial Intelligence
AR	Augmented Reality
CKD	Chronic Kidney Disease
CNN	Convolutional Neural Network
DB	Database
DR	Diet Recommendation
DT	Data Type
FCDB	Food Composition Database
FEA	Food–Effect Analysis
GAN	Generative Adversarial Network
GenAI	Generative Artificial Intelligence
H	High
HCP	Healthcare Professional
K&DF	Knowledge and Data Foundations
KG	Knowledge Graph
L	Low
LLM	Large Language Model
LoTR	Lack of transparency
M	Moderate
MAS	Multi-Agentic Systems
OCR	Optical Character Recognition
PN	Precision Nutrition
RAG	Retrieval-Augmented Generation
RoB	Risk of Bias
SPC	Security and Privacy Concerns
T2D	Type 2 Diabetes
SHAP	Shapley Additive Explanations
USDA	United States Department of Agriculture
VAE	Variational Autoencoder
VS	Validation Source
WGAN-GP	Wasserstein GAN with Gradient Penalty
